# Pravastatin inhibits cell proliferation and increased MAT1A expression in hepatocarcinoma cells and in vivo models

**DOI:** 10.1186/1475-2867-12-5

**Published:** 2012-02-21

**Authors:** Elizabeth Hijona, Jesús María Banales, Lander Hijona, Juan Francisco Medina, Juan Arenas, Marta Herreros-Villanueva, Pablo Aldazabal, Luis Bujanda

**Affiliations:** 1 Department of Gastroenterology, Donostia Hospital, Instituto Biodonostia, University of the Basque Country EHU/UPV, Ciberehd, San Sebastián, Spain; 2 Department of Hepatology, University of Navarra, CIMA, Ciberehd, Pamplona, Spain

**Keywords:** Pravastatin, Sorafenib, Hepatocarcinoma, Statins

## Abstract

**Background:**

Statins may have therapeutic effects on hepatocarcinoma (HCC). This type of disorder is the most common malignant primary tumour in the liver. Our objective was to determine whether pravastatin had a therapeutic effect in vitro and in vivo models.

**Method:**

We design in vitro and in vivo model. In vitro we used PLC and determine cell proliferation. In vivo, we used and animal model to determined, PCNA and MAT1A expression and transaminases levels.

**Results:**

We found that pravastatin decreases cell proliferation in vitro (cell proliferation in pravastatin group was 82%, in sorafenib group 51% and in combined group 40%) and in vivo (in pravastatin group 80%, in sorafenib group 76.4% and in combined group 72.72%). The MAT1A levels, was significantly higher in Pravastatin group (D 62%, P 94%, S 71%, P + S 91%). The transaminases levels, decreased significantly in Pravastatin group (GOT and GPT levels D 619.5 U/L; 271 U/L) (P 117.5 U/L; 43.5 U/L) (S 147 U/L; 59 U/L) (P + S 142 U/L; 59 U/L).

**Conclusion:**

The combination of pravastatin + sorafenib were more effective than Sorafenib alone.

## Background

Hepatocellular carcinoma (HCC) is the fifth most common malignancy worldwide. It ranks third place in the list of malignancies leading to death [1] and the incidence of HCC has increased in eastern Asia, Europe and the United States [2]. Clinically, HCC is characterized by its invasiveness, poor prognosis and limited therapeutic opportunities. In many patients, HCC is diagnosed at an advanced stage. For these patients, the US Food and Drug Administration has approved the multikinase inhibitor, sorafenib [3,4]. In recent years, two studies have been published [5,6] which demonstrate that pravastatin increases the survival of patients with advanced hepatocellular cancer alone or in combination with chemoembolization.

The molecular pathogenesis of HCC is complex and involves the abnormal clonal expansion of dysplastic hepatocytes, anti-apoptotic signalling and the stimulation of angiogenesis-associated growth factors [7].

Today, statins are regarded as attractive molecules and they may affect cancer. Statins, the 3-hydroxy-3-methylglutaryl coenzyme A (HMG-CoA) reductase inhibitors, are a class of drugs that inhibit the rate-limiting step in the cholesterol biosynthesis pathway, cholesterol being an important structural component of cell membranes. Various studies have been reported describing an association of statins with either an increase or a decrease in the incidence of various cancers [8,9]. On the other hand, drug resistance is the major problem of chemotherapy, which causes treatment failure leading to progressive disease. Potential mechanisms of resistance include activation of the Ras/Raf/MEK/ERK signal transduction cascade [10] but also increased cholesterol levels in cancer cells [11].

One of the potential mechanisms of action of statins is the modulation of the cell cycle through the down-regulation of cell cycle promoters such as cyclin D1-dependant kinase (CdK) and the up-regulation of cell cycle inhibitors p21 and p27 [12-15]. It has also been observed that they favour the regulation of homeostasis of the liver by increasing the expression of methionine adenosyltransferase (MAT-1) and decrease cell proliferation by reducing the levels of the Proliferating Cell Nuclear Antigen (PCNA) [16-18]. They also inhibit the activity of matrix metalloproteinases (MMPs), especially of MMP-2 and MMP-9. Further, it has been reported that statins decrease the activity of MMP-9 by 75% [15]. This activity is directly related to tumour invasion and metastasis.

## Materials and methods

### Cell line and culture

The human hepatoma PLC cells were obtained from the ATCC. PLCs were cultured in Dulbecco's modified Eagle's medium (DMEM) supplemented with 10% foetal bovine serum, penicillin G (100 U/ml) and streptomycin (100 μg/ml).

### Cell proliferation

Proliferation in cell culture was measured using the CellTiter 96 AQueous Non-radioactive cell proliferation assay (Promega, Spain). PLC cells were seeded onto 96-well plates, 10,000 cells/ml, and treated for 4 h. Following each treatment, 20 μl of dye solution was added to each well and incubated for 4 h. Subsequently, the absorbance was recorded at 490 nm using an ELISA plate reader. The pravastatin and sorafenib were used at concentrations of 50 μM.

### Animal experiments

All animal studies were performed in accordance with and approved by the Institutional Animal Care and Use Committee. We used Wistar male rats (Charles River, Spain) with initial body weights between 225 and 250 g.

**Figure 1:**
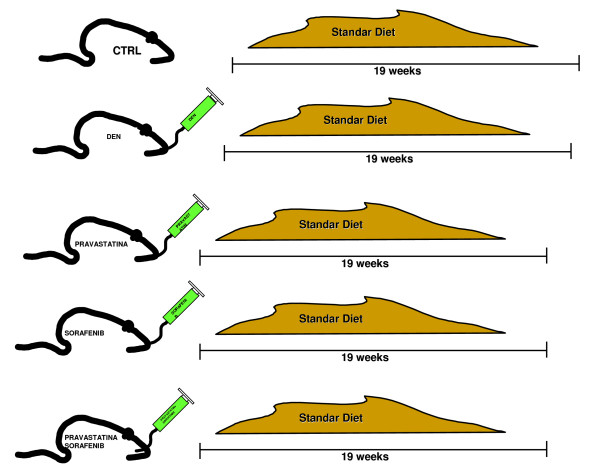
**Animal model**.

To develop a rat model of HCC, we used diethylnitrosamine (DEN). This was administered by orogastric catheter, three times a week for 19 weeks. All these animals developed HCC. The rats were divided into five groups with three experimental groups, corresponding to different drug regimens (Figure 1):

1. CONTROL (C) Group (N = 10): free access to food and water for 19 weeks

2. HCC (D) Group (N = 10): Idem control group but this group were administered DEN three times a week.

3. PRAVASTATIN (P) Group (N = 10): Idem HCC group, with the addition of a dose of 0.6 mg/kg/d of pravastatin given daily by orogastric catheter for 19 weeks.

4. SORAFENIB (S) Group (N = 10): Idem HCC group, with the addition of a dose of 11.4 mg/kg/d of sorafenib daily by orogastric catheter.

5. SORAFENIB + PRAVASTATIN (P + S) Group (N = 10): Idem HCC group, with the addition of a combined dose of 0.6 mg/kg/d of pravastatin and 11.4 mg/kg/d of sorafenib, administered as for P and S Groups.

### Histology

After sacrificing the animal, the liver was sectioned to allow macroscopic assessment of the hepatic lesions. In addition, several portions (1 cm^3^) of the liver were removed and fixed in 10% formaldehyde for 24 h. Subsequently, this tissue was processed: it was embedded in paraffin and 3 and 5 μm sections taken (Microtome, Leitz) which were fixed on slides and stained with haematoxylin and eosin. Samples were then mounted and examined under an optical microscope to characterise the lesions.

### Determination of PCNA and MAT1A

The expression of PCNA (Cayman Chemical, Madrid) and MAT1A (bioNova científica, Madrid) in the liver tissue was measured using specific antibodies. For this, different tissue biopsies (1 cm^3^) were taken, fixed in 40 g/l of formaldehyde buffer, embedded in paraffin and cut into 4 μm sections. Paraffin was removed with xylene, and samples were dehydrated with alcohol and subsequently used for immunohistochemical analysis. The Dako EnVision System-HRP (DAB) was used for immunohistochemical staining, while quantification was carried out using the Genetix Ariol SL-50 system.

### Statistical analysis

The Chi Square test was used to determine the existence of differences in the qualitative variables between the groups, while for quantitative variables ANOVA and Kruskal-Wallis tests were applied depending on the distribution of variables. Multiple comparisons were carried out using the Tukey and Scheffé tests and/or the Mann Whitney test. A level of significance of p < 0.05 was selected.

## Results

### Pravastatin inhibis proliferation in PLC cells

**Figure 2:**
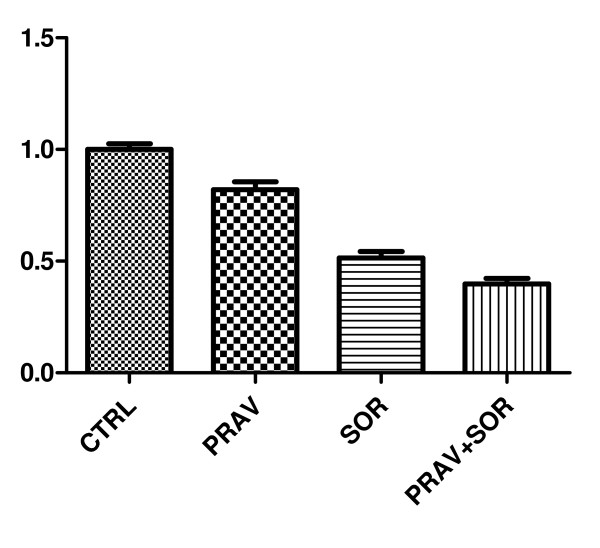
**Levels of proliferation in PLC cells**.

We observed that cell proliferation was considerably lower in all the three experimental groups with the reduction being the most clear in the group administered pravastatin and sorafenib (P 82% (Abs 0.82), S 51% Abs (0.51), P + S 40% (Abs 0.4) compared to the untreated cells; p < 0.05) (Figure 2).

**Figure 3:**
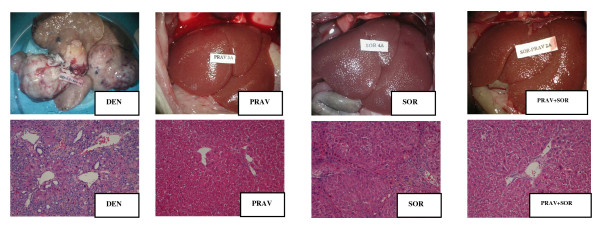
**Macro and microscopic images of livers from each of the groups**.

It was found that all the rats treated with DEN showed advanced HCC at both macro and microscopic levels. In rats treated with pravastatin, sorafenib or a combination thereof, the size of the tumours was significantly smaller and, at the microscopic level, the rats treated with pravastatin and pravastatin + sorafenib contained a smaller number of tumour cells (p < 0.05) while the sorafenib group had dysplastic nodules (Figure 3).

**Figure 4:**
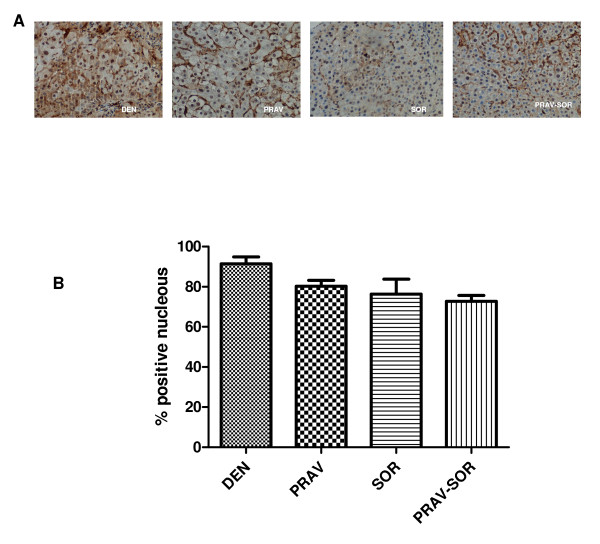
**Expression of PCNA in the different groups**.

In the immunohistochemistry images, we note that the DEN group can be seen to have significantly higher PCNA levels than the other groups (Figure 4A). In the combined P + S Group levels were less elevated than in the other experimental groups (D 91.42%, P 80%, S 76.41%, P + S 72.72%) (Figure 4B)(p < 0.001).

**Figure 5:**
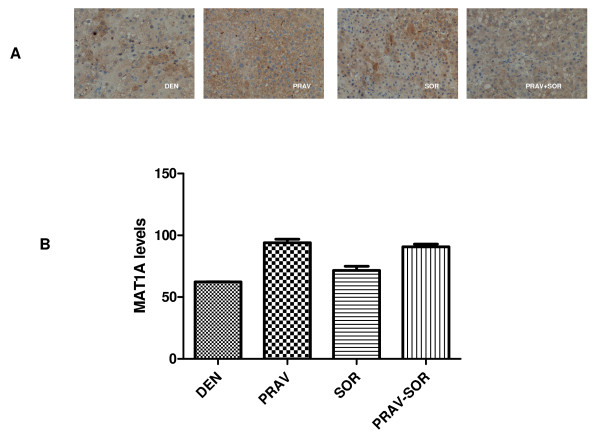
**Expression of MAT1A in the different groups**.

The immunohistochemistry analysis revealed that the level of MAT1A was notably lower in the DEN group, while in the other groups it was significantly higher (p < 0.05) (Figure 5A). This difference was much more significant in the groups given pravastatin than in the other animals (D 62%, P 94%, S 71%, P + S 91%) (p < 0.05) (Figure 5B).

### Pravastatin, sorafenib and the combination of thereof decrease the levels of transaminases

**Figure 6:**
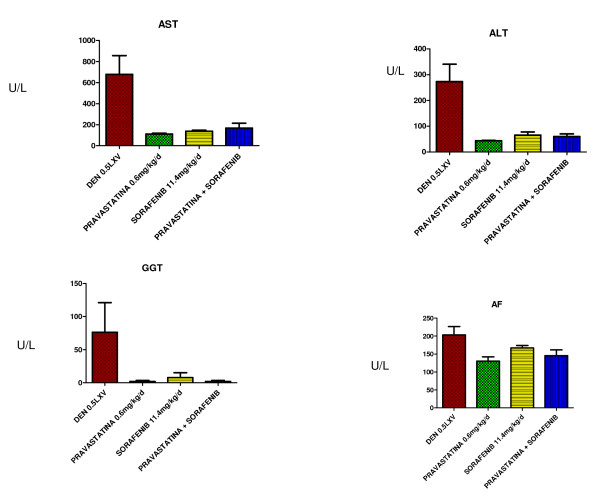
**Levels of AST, ALT, GGT and ALP in the different groups**.

We also observed significantly lower levels of GOT, GPT, GGT and alkaline phosphatise (ALP) in the three experimental groups. Moreover, this decrease was more significant in the pravastatin groups (D 619.5 U/L; 271 U/L; 58.35 U/L; 190 U/L) (P 117.5 U/L;43.5 U/L;7 U/L;129 U/L) (S 147 U/L;59 U/L;23 U/L;172 U/L) (P + S 142 U/L;59.5 U/L;7 U/L;137 U/L) (p < 0.05) (Figure 6).

## Discussion

In spite of the promising results in the treatment of HCC, it still is a disease with poor prognosis, therefore it is necessary to search for new drugs. Moreover, the use of chemotherapy in patients suffering from chronic liver disease is associated with a high rate of severe adverse effects and even death directly linked to treatment. Experimental [18] and epidemiology [19] data have suggested that 3-hydroxy-3-methylglutaryl coenzyme A reductase inhibitors (statins) may have potential as chemopreventive agents in cancer. Observational data have also indicated that statins may have protective effects against the development of cancer, for example, modifying the risk of oesophageal adenocarcinoma in patients with existing Barrett's oesophagus [20]. Two studies [5,6] published in recent years have shown that pravastatin improved survival of patients with advanced hepatocarcinoma. In our study, we found that pravastatin decreases the proliferation of hepatocellular carcinoma cell lines. This finding was then confirmed in a rat model of hepatocarcinoma. Specifically, it was observed that the number of nodules of hepatocarcinoma was lower in rats treated with pravastatin. This small number was also associated with a relatively lower level of serum transaminases.

The role of statins does extend beyond their lipid-lowering effects, as they are known to improve endothelial function, participate in plaque stabilisation, immunomodulation, antioxidant activity, and also act as anti-inflammatory and anticancer agents. These properties have made statins particularly attractive drugs [21]. In this study, we observed that pravastatin decreased the proliferation of hepatocellular carcinoma cell lines. Immunohistochemical staining of proliferating cell nuclear antigen (PCNA) was notably lower in pravastatin group. Expression of proliferating cell nuclear antigen by cells during the S and G2 phases of the cell cycle makes the protein a good cell-proliferation marker [22]. This protein is located in the nucleus and favours the synthesis of DNA.

Another mechanism of action of pravastatin is to cause a decrease in the expression of Methionine Adenosyltransferase (MAT). MAT is the enzyme that catalyzes the synthesis of S-adenosylmethionine (AdoMet), the main donor of methyl groups in the cell [23]. In mammals MAT is the product of two genes, *MAT1A *and *MAT2A. MAT1A *promoter is hypomethylated in liver and hypermethylated in kidney and foetal rat hepatocytes, indicating that this modification is tissue specific and developmentally regulated. Southern blot analysis with a *MAT1A *promoter probe demonstrated that *MAT1A *expression is linked to elevated levels of chromatin acetylation. Early changes in *MAT1A *methylation are already observed in precancerous cirrhotic livers from rats, in which *MAT1A *expression is low. This expression is reactivated in the human hepatoma cell line HepG2 treated with 5-aza-2'-deoxycytidine or the histone deacetylase inhibitor trichostatin, suggesting a role for DNA hypermethylation and histone deacetylation in *MAT1A *silencing [23]. We observed a significant increase in the expression of MAT1A, suggesting that pravastatin has a protective effect against tumour progression.

The inhibition of the products derived from mevalonate may be another mechanism by which pravastatin affects cell proliferation, differentiation and apoptosis. Other authors [18] have reported how the statins inhibit proliferation and induce apoptosis in oesophageal adenocarcinoma cells via inhibition of Ras farnesylation and inhibition of the extracellular signal-regulated kinases and Akt signalling pathways. Pravastatin reduced viable cell numbers and inhibited proliferation in a similar dose-dependent manner. Statins induced apoptosis and enhanced the antiproliferative effect of NS-398, a selective cyclooxygenase (COX)-2 inhibitor, while statin treatment also increased messenger RNA (mRNA) and protein expression of the proapoptotic proteins Bax and Bad. Recently, it has also been observed that pravastatin treatment effectively inhibited the production of several pro-inflammatory/pro-angiogenic mediators involved in inflammation and angiogenesis in vitro studies [24].

Sorafenib, a multikinase inhibitor, increases survival of patients with advanced hepatocellular carcinoma [25]. In one study, median overall survival was 10.7 months in the sorafenib group and 7.9 months in the placebo group [26]. For this reason, one of our objectives was to compare the effectiveness in vitro and in vivo of pravastatin for the treatment of hepatocarcinoma. We observed that the combination of pravastatin and sorafenib in vitro, considerably decreased cell proliferation and the expression of MAT1A in vivo. The results were confirmed in vivo. In particular, the combination of pravastatin and sorafenib resulted in a smaller number and size of hepatocarcinoma lesions, compared to the administration of the two drugs separately. As well as decreasing levels of PCNA and MAT1A, sorafenib also decreased the expression of Mcl-1 messenger RNA and protein, transcriptional targets of STAT3, as well as sensitizing neoplastic cells to tumour necrosis factor-related apoptosis-inducing ligand (TRAIL)-mediated apoptosis [26]. In addition, sorafenib produces inhibition of the expression of phospho-MEK, phospho-ERK, cyclin D1, Rb and anti-apoptotic proteins Bcl-xl and Mcl-1 [27,28]. These facts open new doors for combination treatments for advanced hepatocarcinoma. The great cumulative experience in the use of statins in clinical practice is a very considerable advantage, facilitating the development of clinical trials to assess any increase in survival when combining these two drugs.

The most severe limitation of this study is that we cannot know whether the response in humans would be the same as in in vitro and in vivo models. Further, it is not possible to assess whether or not the adverse effects may strengthen with the use of both drugs.

To conclude, we observe that pravastatin, alone or in combination with sorafenib has substantial antiproliferative effects in hepatocellular carcinoma cell lines in in vitro and in vivo models of hepatocarcinoma. Studies on humans are required to confirm these findings. Pravastatin decreases cell proliferation in in vitro and in vivo models, the combination of pravastatin and sorafenib being more effective than the administration of sorafenib alone.

## Competing interests

The authors declare that they have no competing interests.

## Authors' contributions

EH, LH and LB have designed the project. In vitro determination have been performed by JMB, JFM, MH and EH. In vivo determination have been performed by LH, PA, JA, LH and EH. Histology study have been performed by LH, JMB, PA and LH. Statistical study have been performed by LH, LB and EH. All authors write the manuscript. All authors read and approved the final manuscript.

### Grant support

In addition, this work was supported by grants from the Department of Health of the Basque Government 2009/111003.
